# Evaluating the effect of an educational program on increasing cervical cancer screening behavior among women in Fasa, Iran

**DOI:** 10.1186/s12905-021-01191-x

**Published:** 2021-01-28

**Authors:** Maryam Heidari Sarvestani, Ali Khani Jeihooni, Zahra Moradi, Azizallah Dehghan

**Affiliations:** 1 Shiraz University of Medical Sciences, Shiraz, Iran; 2 Department of Public Health, School of Health, Shiraz University of Medical Sciences, Shiraz, Iran; 3 Department of Nursing, School of Nursing, Fasa University of Medical Sciences, Fasa, Iran; 4 No Communicable Diseases Research Center, Fasa University of Medical Sciences, Fasa, Iran

**Keywords:** Pap smear, The theory of planned behavior, Women

## Abstract

**Background:**

Cervical cancer is the second most common cancer and the fifth deadliest cancer among women in Iran. Educational interventions based on the proper behavior promoting models can lead to early diagnosis of cervical cancer.This study aimed to investigate the effects of educational intervention on performing Pap smear tests based on the Theory of Planned Behavior among women living in Fasa, Iran.

**Methods:**

A cross-sectional study was conducted on 700 participants. Thereafter, the educational intervention based on the results of cross-sectional study was conducted in a workshop form for 50 women as the intervention group and 50 women as the control group. Afterward, the data were entered into the SPSS statistical software and then analyzed via logistic regressions analysis, paired *t* test, independent *t* test, chi-square test, and McNemar test.

**Result:**

According to the results, 45.7% of the patients had a history of performing a Pap smear test, and 20.7% of them regularly performed this test. The knowledge, attitude, subjective norms, and perceived behavioral control were the predictors of intention and behavior of Pap smear test among the women (*P* < 0.05). These components accounted for 57.4% and 31.6% of the intention and behavior variances, respectively. After the intervention, a significant increase was observed in the means of attitude, subjective norms, and perceived behavioral control in the intervention group compared to the control group. The results reveal no significant difference between these two groups regarding the behavioral intention (*p* = 0.41) and performance of the Pap smear test (*p* = 0.583). The number of the participants undergone the Pap smear test has increased from 10 to 26 in the intervention group by passing 3 months from the intervention. The results of McNemar test indicated that this difference was statistically significant.

**Conclusion:**

The results indicated an increase in the women’s performance of the Pap smear screening test by appropriate planning, provision of educational packages based on the women’s needs, and using effective subjective norms.

*Trial Registration*: Current Controlled Trials IRCT20160830029608N3:12/31/2018. “Retrospectively registered”.

## Background

Cervical cancer is the second most common cancer and the fifth deadliest cancer among women in Iran [1]. Moreover, it is the most common cancer in developing countries. Annually, one out of every 123 women is infected with cervical cancer, with the mortality rate of 1 per 100/000 [2]. The World Health Organization (WHO) has estimated that cervical cancer will be responsible for 474/000 deaths up to 2030, with 95% of the deaths in low- and middle-income countries [3]. Nonetheless, considering the long pre-invasion period, accessibility of appropriate screening programs, and effective treatment of the primary lesions, cervical cancer is known as a preventable cancer [3]. By the utilization of Pap smear test as a screening technique, the prevalence of cervical cancer has reduced by 79% up to now [4]. Therefore, performance of this test should be considered as a health policy for those sexually active women [5]. Benedet et al. [6] maintained that the women undergone the Pap smear test more than 3 years ago and those who had not undergone the test at all, were at the highest risk for cervical cancer.

Educational interventions based on the proper behavior promoting models can lead to early diagnosis of cervical cancer, which eventually decreases the resultant mortality. Among the models used for the perception and prediction of health behaviors, the theory of planned behavior has been successful. The theory of reasoned action has also been designed to predict and explain behaviors based on the assumption that individuals select their behaviors in terms of the logical investigation of the available information and by considering the results of their performance prior to decision-making. According to this theory, the best predictor of behavior is the individual’s intention for his/her involvement in the behavior. Correspondingly, intention is predicted by two variables; first, attitude related to the behavior (overall positive or negative evaluation of the behavior), and second, subjective norms (overall perception of social pressure to do the behavior). The rate of success of this theory in explaining the behavior mostly depends on the extent the behavior is controlled by the individual. In fact, individuals normally act based on their perception of how others think about what they should do. Besides, their intention to accept the behavior is potentially affected by those individuals with whom they have close relationships. In this theory, subjective norms are obtained through multiplying normative beliefs by the motivation of performing the target behavior, in contrast with the expectations. In addition, perceived behavioral control has been defined as the extent to which an individual believes about the controllability of a behavior [7–10]. In the present study, the theory of planned behavior was selected to create and improve the Pap smear screening behavior among women in Fasa city. This model has been employed in numerous studies. For instance, Matlabi et al. [11] in their study conducted on 140 married women aged between 20 and 49 years old to explore the impact of education on the promotion of breast self-examination using the stages of change model. The results revealed a significant increase in the means of knowledge, attitude, perceived behavioral control, behavioral intention, and breast self-examination behavior in the intervention group after the educational intervention. However, no significant changes were observed in the control group. These results confirmed the effectiveness of the educational intervention on the promotion of breast self-examination behavior as a screening method for breast cancer [11]. Similarly, Taghdisi et al. (2016) assessed the effect of an educational intervention on the improvement of the consumption of fruits and vegetables among students based on a behavioral theory. The results showed an increase in the intervention group’s knowledge, attitude, perceived behavioral control, and subjective norms compared to the control group after the intervention, which consequently promoted the consumption of fruits and vegetables [12]. In the same vein, Jeihooni et al. [13], based on a behavioral theory, evaluated the effect of an educational intervention on the performance of mammography as a breast cancer screening technique. The results indicated a significant increase in the intervention group’s knowledge, attitude, perceived behavioral control, and subjective norms compared to the control group by passing 6 months from the intervention. After the intervention, 78% of the women in the intervention group were willing to perform mammography and 74% finally did that. Accordingly, these measures were obtained as 19% and 7% in the control group, respectively. Hence, the educational intervention based on the behavioral theory was effective on the improvement of the performance of mammography among the women [13]. The theory of planned behavior pays attention to social factors and motivation for following the significant ones. Hence, various studies have found it as an important factor in the acceptance of desirable behaviors like performing the Pap smear test [14, 15]. Considering the importance of Pap smear screening for early detection of cancer cervix in women, the need for educational intervention to increase participation in performing this test, and due to the fact that no intervention study has been conducted in this regard in Fasa city so far, the present study aimed to investigate the effect of an educational program on increasing cervical cancer screening behavior based on the Theory of Planned Behavior (TPB) among women in Fasa city, Fars province, Iran.

## Methods

### Design

This descriptive-analytical, cross-sectional study was conducted in Fasa in 2016.

### Sample and setting

Of six healthcare centers in Fasa, two centers were selected through simple random sampling. Thereafter, based on the lists of the households covered by each center, 350 people were selected from each center via simple random sampling (700 people in total) who were then enrolled into the research. During the sampling, two researcher’s assistants were present in the healthcare centers, provided the eligible women with the necessary information, invited them to take part in the research, and finally obtained their written informed consent forms. The inclusion criteria of the study were the followings: being married, age above 21 years old, married at least 3 years ago, having no history of cancer and hysterectomy, and not having performing the Pap smear test during the past 3 years. The exclusion criterion of the study was the absence in the 3-month follow-up.

**Fig. 1:**
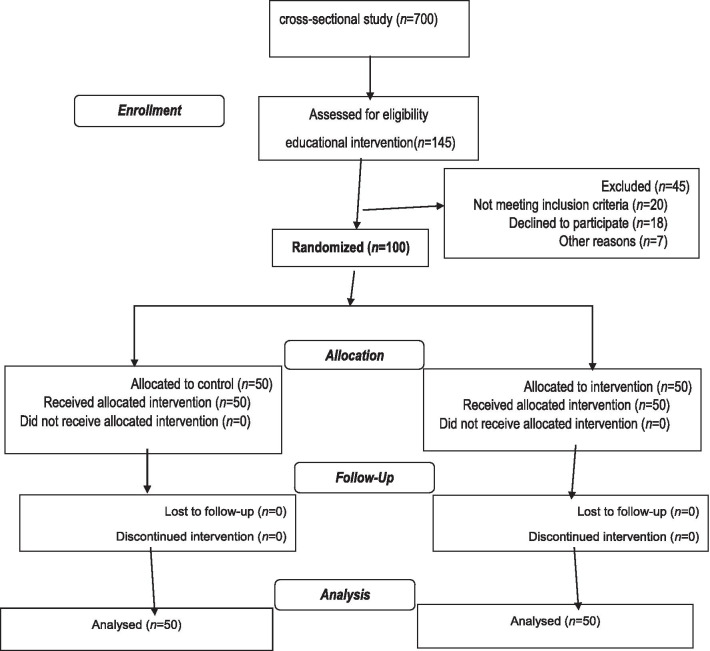
Flow chart of study

At first, a cross-sectional study was conducted on 700 participants [16]. Subsequently, they were requested to fill out the demographic information and theory of planned behavior questionnaires. The obtained results were analyzed and the educational intervention was designed. Next, the educational intervention was conducted in a workshop form for 50 women as the intervention group and 50 women as the control group (Fig. 1 shows the study flowchart).

### Measures

The study data were collected using an instrument including three sections, which was prepared based on the Moradi et al. [16] and Roncancio et al. [17] studies that its validity and reliability were confirmed. The first part of the questionnaire contained demographic information such as age, number of children, age at the time of the first pregnancy, history of performance of the Pap smear test, occupation, educational level, menopause status, and family history of cervical cancer. The second and third parts of the questionnaire consisted of some questions on knowledge and the constructs of the theory of planned behavior. In this questionnaire, knowledge was assessed by 15 yes/no questions (yes = 1, no = 0). in addition, attitude was evaluated by six items (e.g. Annually performing the Pap smear test reduces the probability of cervical cancer) responded via a five-option Likert scale ranged from completely disagree [1] to completely agree [5]. Moreover, subjective norms of the persuasive person for performance of the Pap smear test were determined by five items (e.g. My husband persuades me to perform the Pap smear test on a regular basis) responded through a five-option Likert scale ranged from very low [1] to very high [5]. Besides, perceived behavioral control was measured by two items (e.g. Performance of the Pap smear test is difficult for me) responded using a five-point Likert scale ranged from very low [1] to very high [5]. Intention to perform the Pap smear test was also evaluated using two items (e.g. I intend to perform the Pap smear test this year) responded via a five-option Likert scale ranged from very low [1] to very high [5]. Finally, screening behaviors and performance of the Pap smear test were both assessed via two items whose scores ranged from 2 to 18. The content validity of the questionnaire was assessed by 18 health education specialists, one MSc of midwifery, and one gynecologist. Considering Content Validity Ratio (CVR) and Content Validity Index (CVI), the necessary modifications were applied and the indices were then confirmed. Moreover, a pilot study was conducted on 30 eligible women. Notably, the reliability coefficients of attitude, subjective norms, behavioral intention, and perceived behavioral control constructs were found to be 0.74, 0.75, 0.65, and 0.76 respectively.

Subsequently, the study data were entered into the SPSS 22 software and then analyzed using Pearson’s correlation coefficient and logistic regression analysis. *P* < 0.05 was considered as statistically significant.

### Intervention

The educational intervention was conducted through lecture, group discussion, question and answer, educational pamphlets and posters, movie, and PowerPoint in form of three 45-min sessions. Accordingly, these educational sessions were held once a week. In the third session, physicians, healthcare staff, and the participants’ husbands were present as subjective norms. The educational content included cervical cancer, importance of performing screening behaviors, probable obstacles against the performance of the Pap smear test, and strategies for overcoming individual and environmental barriers. The intervention was performed through face-to-face training, group discussion (about beliefs, positive and negative outcomes of the behavior, facilitators, motivation to follow the significant ones, and subjective norms), educational pamphlets, and PowerPoint. In addition, an educational movie on how the Pap smear test is done, was shown for the participants. The discussions were directed towards the identification of positive beliefs and attitudes, in order to promote positive motivation for performing diagnostic measures, particularly the Pap smear test, and to provide the ground for development of a positive attitude or modification of the negative attitude on the uncontrollability of the disease. Moreover, some wrong beliefs about the Pap smear test that are common were put under debate, in order to reduce the intensity of wrong beliefs by modifying the participants’ beliefs and attitudes. At the end of the educational sessions, the participants were provided with an educational booklet. Besides, a WhatsApp group was created for information’s sharing. An informed SMS was also sent to the participants once a week. However, in the control group, the participants were provided only with the educational booklet.

### Statistical analysis

All the participants were followed up until 3 months after the educational intervention and the theory of planned behavior questionnaire was completed for them once again. Furthermore, they were required to send the results of their Pap smear tests to the researcher in case that they performed the test. Afterward, the data were entered into the SPSS statistical software and then analyzed via logistic regressions analysis, paired *t* test, independent *t* test, chi-square test, and McNemar test.

### Ethics

This study was approved by the Research Council (code: 95,094) and Ethics Committee (IR.FUMS.REC.1396.187) of Fasa University of Medical Sciences. It was also registered in the Iranian Registry of Clinical Trials (IRCT20160830029608N3).

## Results

In the present research, 100 women (50 subjects for the experimental group and 50 subjects for the control group) were investigated under cover of health centers of Fasa city. None of the cases was excluded from the intervention and control groups. The average age of experimental group was 10.17 ± 35.62 years old and average age of the control group was 9.55 ± 34.12 years old. Notably, based on the result of independent *t* test, no significant differences were found between the experimental and control groups (*P* = 0.154). Additionally, demographic characteristics of the subjects were not significantly different (Table 1).

**Table 1:** Comparison of qualitative variables in two groups of intervention and control women

Variable	Group		
	Case (n = 50)	Control (n = 50)	*p* value
	Mean ± SD	Mean ± SD	
Age	35.62 ± 10.17	34.12 ± 9.55	0.154
Education			0.253
Elementary	6 (12)	5 (10)	
Intermediate	25 (50)	23 (46)	
High school	18 (36)	20 (40)	
Collegiate	1 (2)	2 (4)	
Job			0.345
Housewife	44 (88)	45 (90)	
Employed	6 (12)	5 (10)	

Numbers in parentheses show percent

The results reveal that, before educational intervention, there were no significant differences between the experimental and control groups in knowledge, attitude, subjective norms, and perceived behavioral control. However, by passing 3 months from the intervention, the experimental group showed significant enhancement in each one of the above-mentioned factors in which the experimental group indicated more significant reduction compared to the control group (Table 2).

**Table 2:** Comparison of mean scores of planned behavior theory in the two groups before and 3 months after the educational intervention

TPB constructs	Before the intervention	Three months after the intervention	*p* value
Attitude			
Control	3.34 ± 20.25	3.28 ± 20.6	0.19
Case	3.25 ± 20.68	2.35 ± 25.58	*P* < 0/001
*p* value	0.18	*P* < 0/001	
Subjective norms			
Control	3.60 ± 14.14	3.45 ± 14.21	0.23
Case	3.64 ± 14.18	3.28 ± 18.33	*P* < 0.001
*p* value	0.68	*P* < 0.001	
Perceived behavioral control			
Control	1.00 ± 5.74	1.12 ± 5.80	0.35
Case	1.00 ± 5.82	2.11 ± 8.89	*P* < 0.001
*P* value	0.65	*P* < 0.001	
Control	2.14 ± 5.78	2.31 ± 5.85	0.41
Case	2.35 ± 5.34	2.12 ± 9.45	*P* < 0.001
*P *value	0.76	*P* < 0.001	
Behavioral intent			
Control	3.34 ± 20.25	3.28 ± 20.6	0.19
Case	3.25 ± 20.68	2.35 ± 25.58	*P* < 0/001
*P* value	0.18	*P* < 0/001	

The results reveal no significant difference between these two groups concerning the behavioral intention (*p* = 0.41) and performance of the Pap smear test (*p* = 0.583) (Tables 2, 3). The number of the participants undergone the Pap smear test increased from 10 to 26 in the intervention group by passing 3 months from the intervention. Accordingly, the results of McNemar test indicated that this difference was statistically significant (*p* < 0.001, Table 3).

**Table 3:** Frequency distribution of Pap smear test before and after the educational intervention

Variable	Case (n = 50)	Control (n = 50)	*p* value
Before the intervention			
Pap smear	10 (20)	8 (16)	*P* = 0.583
Failure to do pap	40 (80)	42 (84)	
After the intervention			
Pap smear	26 (52)	10 (20)	*P* < 0/001
Failure to do pap	24 (48)	40 (80)	

Numbers in parentheses show percent

## Discussion

Numerous studies have demonstrated that the most important reasons for not performing the Pap smear test are insufficient education, lack of knowledge on cervical cancer and the Pap smear test, negative attitude towards examination, cultural problems, incorrect perception of the disease, psychosocial factors, and demographic features. Considering the women’s low knowledge level and inappropriate performance in this regard, proper training and interventions seem to play pivotal roles in enhancing their knowledge level and promoting their participation in the Pap smear test to prevent cervical cancer [18, 19].

Bahmani et al. in their study, based on the precaution adoption process model, investigated the effect of an educational intervention on the cervical cancer screening behavior. The results revealed the effectiveness of this model on the improvement of the cervical cancer screening behavior. Although the utilized educational intervention was different from the one used in the present study, the results of both studies indicated that educational interventions could promote health-related behaviors in the community [20]. Hazavehei et al. (2016) in a systematic review have also explored the impact of educational interventions on enhancing Pap smear screening. Among the investigated articles, seven of them were conducted using health education theories and models, while five of them were performed without the use of these theories and models. The findings demonstrated that the utilization of multifaceted educational methods as well as the application of health education and health promotion theories and models are highly effective on the improvement of individuals’ behavioral performances. As mentioned earlier, health education and health promotion interventions play critical roles in the elimination of problems and in the promotion of behaviors like performance of the Pap smear test. When these interventions are accomplished based on models and theories, finding the constructs under the influence of the theories and models can help in designing interventions for the promotion of the behavior. This can, in turn, play a key role in the evaluation and documentation of change strategies [18].

The results of a meta-analysis conducted by Naz et al. revealed the effectiveness of educational interventions based on health behavior change theories on the improvement of women’s behavior regarding the performance of cervical cancer screening worldwide. In this respect, the theory of planned behavior was found to be a beneficial model for determining the women’s tendency to perform cervical cancer screening. Overall, theory-based educational interventions could increase the women’s knowledge in this field and also enhance their intention to perform the cervical cancer screening test [21]. The findings of a meta-analysis by Steinmetz et al. [22] have also proved the effectiveness of the interventions based on the theory of planned behavior on behavior change. These results were in agreement with those of the current investigation.

Ebu et al. [23] assessed the effect of planned interventions based on the health belief model on breast cancer and cervical cancer screening as well as the performance of the Pap smear test among 870 women. Correspondingly, the results indicated an increase in the women’s intention to perform Pap smear test after the intervention. Although the utilized method was different from that employed in the present study, the results confirmed the positive effect of educational interventions on the promotion of cancer prevention methods among women.

Molaei-Zardanjani et al. in their study reported that education based on the theory of planned behavior (either in person or in groups) was effective on the women’s attitude, subjective norms, perceived behavioral control, and behavioral intention regarding the performance of mammography. In fact, the educational intervention improved the breast cancer screening behavior, which was in line with the results of the present study [24].

Karakuş Selçuk and Yanikkerem investigated the effect of education on the women’s attitude and behavior regarding breast cancer and cervical cancer screening. Accordingly, the results showed that 28.4% of the women performed mammography, 69.9% performed breast self-examination, and 33.6% of them performed the Pap smear test 6 months after the intervention. Hence, the training was found to be effective on the promotion of the women’s attitude and behavior regarding the performance of screening. Although the used educational method was not similar to that utilized in the current study, the results indicated the women’s need for a professional educational program, in order to improve their attitude and behavior regarding screening programs [25].

The results of Abamecha et al.’s study showed that the rate of knowledge of the disease signs, symptoms, risk factors, and prevention methods was 162(41.4%). Moreover, knowledge on the disease and past screening experience were positively associated with the intention to use cervical cancer screening. Furthermore, standardized regression coefficient showed that all dimensions of TPB were positively associated with the intention to use the services with perceived behavioral control, perceived social pressure, and attitude towards screening [26].

In Mirzaei-Alavijeh et al.’s study, 3 months after the educational intervention, significant improvements were found in average response for Attitude, subjective norms, perceived behavior control, and behavioral intention toward undergoing Pap smear among the intervention group. Additionally, after the intervention, the rate of intention to perform Pap smear test has increased among the intervention group. This study indicated that the educational program based on theory of planned behavior could encourage women to do Pap smear test regularly [27].

Some of the limitations of this study were self-reporting answers of the included subjects and lack of accurate answers to some questions due to shame feeling. Of course, the subjects were ensured that their information would remain confidential. Considering the fact that sampling in this study was performed from those people referred to health centers and not based on population, the study does not have a good external validity. In addition, the generalization of the results to other places should be done with caution. In this regard, performing population-based studies is recommended. The strong points of the research included the creation of the WhatsApp group, continual repetition of the trainings, and reception of the Pap smear test results besides the women’s report of performance of the test.

## Conclusion

The results of this research indicate that educational intervention based on theory of planned behavior led to the enhancement of knowledge, positive attitude, self-subjective norms, perceived behavioral control, and behavioral intention in the studied subjects, which consequently caused the promotion of performance of the Pap smear test of the studied women. Therefore, for performing the Pap smear test among women, theory of planned behavior is recommended. It is noteworthy that engaging families and health officials such as doctors and health workers in educational programs, providing places for performing Pap smear test for women would have a great effect on performance of the Pap smear test.

## Data Availability

The datasets used and/or analyzed during the current study are available from the corresponding author upon reasonable request.

## Abbreviations

TPB: Theory of planned behavior
CPU: Central Processing unit
